# The Role of Perinatal Cardiology in Saving the Life and Its Quality of Fetuses, Newborns and Children (on the Basis of Own Experience and Review of The Literature)

**DOI:** 10.34763/devperiodmed.20182203.270279

**Published:** 2018-10-04

**Authors:** Maria Respondek-Liberska

**Affiliations:** 1 Department for Prenatal Diagnoses and Prevention, Medical University of Lodz, Lodz, Poland; 2 Department of Prenatal Cardiology, Polish Mother’s Memorial Hospital, Research Institute, Lodz, Poland

**Keywords:** prenatal echocardiography, congenital heart defects, congestive fetal heart failure, fetal therapy, delivery, prenatalna echokardiografia, wada serca, niewydolność krążenia, terapia, poród

## Abstract

The role of prenatal cardiology and the organization of perinatal cardiological centers in early diagnostics and early therapeutic procedures in fetuses and newborns with cardiac malformations and circulatory disturbances was discussed on the basis of the literature and own experience. The possibilities of an early perinatal diagnosis and early therapeutic approaches to cardiac defects were presented. It was stressed that there is a necessity to broaden the educational aims in these areas and in the near future to prepare multidisciplinary teams working together in specialist centers.

## Streszczenie

Na podstawie doświadczeń własnych oraz danych z piśmiennictwa przedstawiono rolę kardiologii prenatalnej i jej znaczenie w ośrodkach referencyjnych dla wczesnej diagnostyki i zabiegów terapeutycznych u płodów i noworodków z wadami wrodzonymi serca oraz anomaliami w zakresie układu krążenia. Przedstawiono podstawy i możliwości wczesnej diagnostyki kardiologicznej i zasad prowadzenia terapii. Podkreślono również konieczność poszerzenia procesu edukacji w celu przygotowania w niedalekiej przyszłości medycznych zespołów interdyscyplinarnych do pracy w płodowo/perinatalnych centrach szpitalnych.

## Introduction

The recent huge medical progress enables us not only to save life but also to ensure its high quality. The major role in making this possible is played by the early diagnosis of a disease and an early therapeutic approach. To obtain this goal in fetuses and neonates, prenatal care and perinatal centers are necessary.

**Fig. 1:**
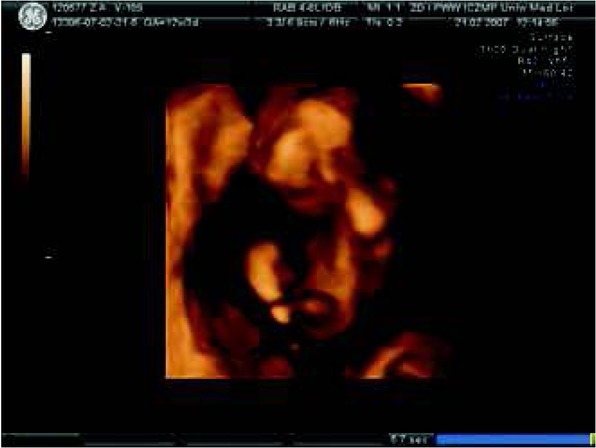
Fetal body in 3D ultrasound at the 12th week of gestation – one of the very first medical diagnostic imaging examinations. Ryc. 1. Zarysy całego płodu w badaniu USG w prezentacji 3D w 12 tyg. ciąży – jedno z pierwszych medycznych badań diagnostycznych.

The majority of human malformations arise between the 4th and 6th week of pregnancy [1]. At the 8th week of pregnancy, embryogenesis is finished. Currently, the first prenatal examination of fetuses is possible around the 12th week of pregnancy (Figure 1) (Earlier scans at 4-6-8 weeks are performed only to confirm the presence of pregnancy, its location and whether it is single or multiple).

Some 10-15 years ago a rule was introduced by Professor M. Hansmann, founder of the German Prenatal Ultrasonography School, that the first sonographic examination should be performed at the 10th week of pregnancy, which was widely used [2]. In contrast, Professor K. Nicolaides, founder of the Fetal Medicine Foundation, suggested that delaying the first sonographic examination until the 12th week of gestation may provide more data on the development and structure of the fetus than that performed 2 weeks earlier [3].

Since the 10^th^-12^th^ week of gestation different sonographic techniques i.e. transvaginal and transabdominal examination of the fetus along with maternal blood tests may provide information about the pregnancy (meaning the fetus’s) development. The woman’s health during pregnancy is under separate – different control.

The prevalence of congenital malformation is around 1-3%. Most of the screening results of the pregnant women performed in the 1st trimester are negative and then the evaluation of the fetus is repeated at the 18^th^-20^th^ week of pregnancy.

An exception is a pregnancy of “high risk” (for instance, the presence of cardiac malformation in the family). In such cases, fetal cardiac examination should be performed earlier – i.e. in the 13^th^-15^th^ week of pregnancy in a reference center [4]. The so-called “early echocardiography” in the majority of cases confirms normal fetal heart structure and allows the safe continuation of pregnancy, relieving the stress of the pregnant woman. The examination of the fetal heart performed at this stage should be treated as preliminary and it requires further confirmation at later stages.

During the first half of the pregnancy (since the 12th week of gestation) it is also possible to detect severe fetal heart malformations and in such cases some women may decide not to continue their pregnancies [5].

**Fig. 2:**
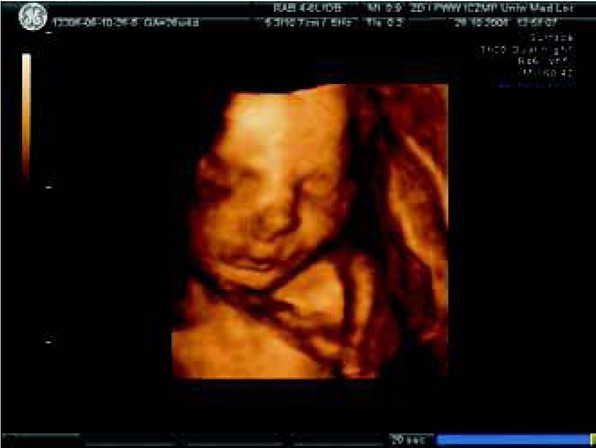
Fetal face in 3D ultrasound at the 26th week of gestation – no dysmorphic feature, however this does not exclude normal or abnormal fetal heart. Ryc. 2. Zarysy twarzy płodu w badaniu USG w prezentacji 3D w 26 tyg. ciąży – nie ma cech dysmorfii, co nie wyklucza obecności wady serca płodu lub prawidłowej budowy serca płodu.

**Fig. 3:**
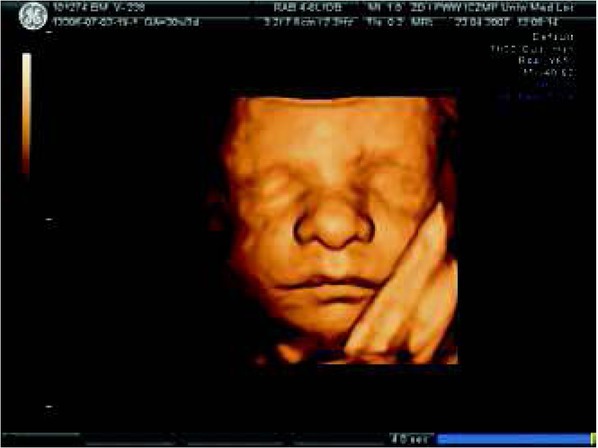
Fetal face in 3D ultrasound at the 30th week of gestation and no dysmorphic features. Patient during fetal echocardiography monitoring his haemodynamic condition due to his heart defect (since embryogenesis.) Ryc. 3. Zarysy twarzy płodu w badaniu USG w prezentacji 3D w 30 tyg. ciąży bez cech dysmorfii. Na tym etapie ciąży, wiedząc, iż u płodu powstała w okresie embriogenezy wada serca prowadzimy monitorowanie echokardiograficzne stanu hemodynamicznego pacjenta.

**Fig. 4:**
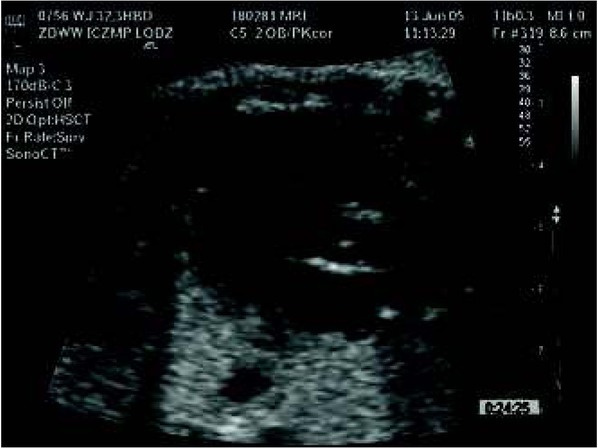
One of the fetal echocardiography scans of the left ventricle in semi long view with chordea tendinea (arrow), which later on might be a cause of the heart murmur during auscultation, despite normal heart anatomy. Ryc. 4. Jeden z przekrojów serca płodu w czasie badania echokardiograficznego ukazujący w świetle lewej komory serca nitkę ścięgnistą (strzałka), która może być przyczyną szmeru u dziecka z prawidłową budową serca.

**Fig. 5:**
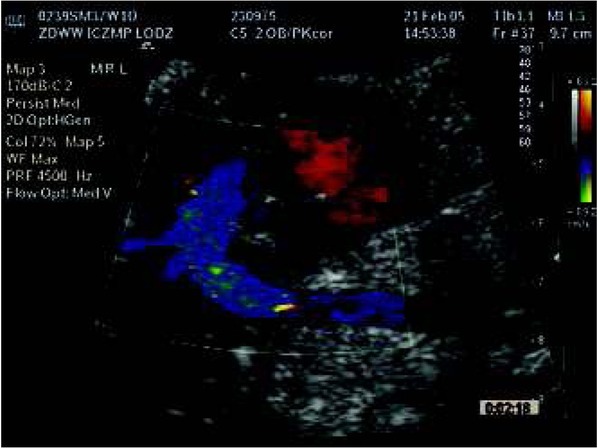
Fetal aortic arch in long axis scan with color Doppler showing normal laminar blood flow. Ryc. 5. Obraz łuku aorty płodu w osi długiej w badaniu echokardiograficznym z kolorowym Dopplerem pokazujący lamilarny kierunek przepływu krwi.

In the middle of pregnancy, an adequately trained physician armored with an “electronic stethoscope” i.e. the appropriate ultrasonographic probe, is able to judge the size and placement of the fetal heart and also the origin of large vessels. However, the fetal heart exam should be performed after the evaluation of the fetal head, face, skeletal system, thorax, limbs, abdomen, fetal and placental umbilical cord attachment. An important step is the evaluation of the fetal heart position (levocardia?, dextrocardia?, mesocardia?, ectopia ?) , its size, axis, 4-chamber view, mediastinum, big vessel relations [6]. Examples of ultrasound exams are presented in some photographs (Figure 2, 3, 4, 5).

The majority of congenital heart malformations may not only be detected at this stage of pregnancy but also precisely diagnosed. In the past, only cardiac pathologists were able to do the latter [7]. This was in the era of the medicine of our “fathers and grandfathers”.

Taking into consideration that the majority of obstetricians do not get a pediatric or cardiac background during their medical training, such detailed cardiac diagnoses should not be expected from their examinations and preliminary diagnoses. It is worth remembering that neither do these doctors have current knowledge about the progress in cardiology or cardiac surgery.

Therefore, the main role of the obstetrician is to detect any cardiac abnormality and then the pregnant woman should be directed to a reference center, where specialized prenatal cardiologists, perinatologists, radiologists and geneticists work. Such a team should be prepared to perform diagnostic examinations and therapeutic procedures, both in common, as well as in complex and rare syndromes. The elements of echocardiographic examination in a referral center (including the fetal heart) are shown in Table I.

**Table I:** Elements of the fetal echocardiography exam in the middle of gestation in a tertiary fetal cardiology center Tabela I. Elementy badania echokardiograficznego u płodu w połowie ciąży w ośrodku kardiologii prenatalnej.

•	Fetal heart position: Levocardia? Dextrocardia? Mesocardia? Ectopia cordis? *Położenie serca: Po lewej stronie klatki piersiowej, Po prawej stronie? Pośrodku? Czy poza klatką piersiową?*
•	Fetal heart size: Normal? Cardiomegaly? Heart too small for gestational age? *Wielkość serca: Norma? Kardiomegalia? Serce za małe w stosunku do wieku płodu*
•	Heart axis: Normal, abnormal (degree) *Oś serca: Prawidłowa, nieprawidłowa, określenie w stopniach*
•	Heart anatomy: Atria: appendages, intraatrial septum, foramen ovale, size, foramen ovale flap, direction of the fetal blood flow, maximal velocity of blood flow, spectral Doppler, flow, sound of the blood flow
	*Budowa serca: Przedsionki: uszka przedsionków, przegroda międzyprzedsionkowa, otwór owalny wielkość, zastawka otworu owalnego, kierunek przepływu przez otwór owalny, prędkość przepływu krwi, spektrum przepływu, dźwięk*
•	Fetal heart valves: atrioventricular, semilunar, opening, movement, position, size, regurgitation? stenosis? maximal blood flow velocity? Doppler blood flow spectrum *Zastawki serca: przedsionkowo-komorowe, półksiężycowate: otwarcie, ruch, położenie, wielkość, niedomykalność? prędkość przepływu? spektrum przepływu?*
•	Fetal heart chamber: size, trabeculation, morphology, contractility, walls thickness *Komory serca: wielkość, trabekulacja, morfologia, kurczliwość, grubość ścian*
•	Big vessels: atrio-ventricular concordance or discordance, size of big vessels, position, direction of the blood flow *Duże naczynia: połączenia przedsionkowo-komorowe zgodne czy niezgodne, szerokość pierścieni naczyniowych, kierunek przebiegu, kierunek przepływu*
•	Aorftc arch: left? Right? Double? Interrupted? Hypoplastic? Narrow aortic isthmus? Maximal blood flow velocity, Doppler spectra, direction of the blood flow *Łuk aorty: lewostronny?, prawostronny?, podwójny?, przerwany?, hipoplastyczny? z wąską cieśnią aorty? Prędkość maksymalna przepływu krwi, spektrum przepływu, kierunek przepływu krwi*
•	Ductus arteriosus: size, shape, position, maximal blood flow velocity, Doppler blood flow spectrum *Przewód tętniczy: szerokość, kształt, położenie, prędkość maksymalna przepływu krwi, spektrum przepływu krwi*
•	Systemic veins: vena cava superior? vena cava inferior? size connection to the right atrium? persistent left superior vena cava? vena azygos *Żyły systemowe: żyła główna górna?, żyła główna dolna? (szerokość? spływ do prawego przedsionka? żyła główna górna lewa? żyła bezimienna?*
•	Pulmonary veins: to left atrium? spectral blood flow velocity? shape? *Żyły płucne: ujście do lewego przedsionka? spektrum przepływu krwi? prędkość maksymalna przepływu krwi?*
•	Systolic function of the right chamber? left chamber? right atrium? left atrium? Funkcja skurczowa prawej komory?, lewej komory?, prawego i lewego przedsionka?
•	Diastolic function of the right and left fetal heart chamber Funkcja rozkurczowa prawej i lewej komory serca płodu?
•	Peripheral blood flow: in ductus venosus, middle cerebral artery, umbilical arteries and vein *Przepływy obwodowe: przewód żylny, tętnica środkowa mózgu, tętnica i żyła pępowinowe*
•	Fetal heart rhythm: frequency? regularity? sinus rhythm? junctional rhythm? atrio-ventricular block? *Rytm serca: częstość? miarowość? rytm zatokowy? węzłowy? blok przewodzenia?*

The prevalence of fetal cardiac malformations varies; according to Norwegian data it is 30/10 000 pregnancies [8] while the Chinese reported prevalence from 19/1000 [9] to 27/1000 [10]. Those differences may reflect the differences in the methodologies used, including whether ultrasound was used by sonographers (in Europe) or it was fetal echocardiographic examination performed by a special team (in China). All the authors agreed, however, that the frequency of fetal cardiac malformations is higher than in newborns.

This is also true in Poland. According to the current Polish law, in cases of severe fetal malformation detected and diagnosed in the first half of pregnancy, or rather until the fetus is able to survive outside the womb, it is the future mother who is in a position to make the decision whether to stop or continue the pregnancy. Terminating the pregnancy means provoking early delivery using prostaglandins on the hospital premises and giving the woman the best chance for the next pregnancy later on.

According to the Polish National Registry of Fetal Cardiac Defects, the majority of pregnant women (over 85%), would like to continue their pregnancies, despite the prenatal diagnosis of heart defect and give the chance of early treatment to the newborn [11].

**Fig. 6:**
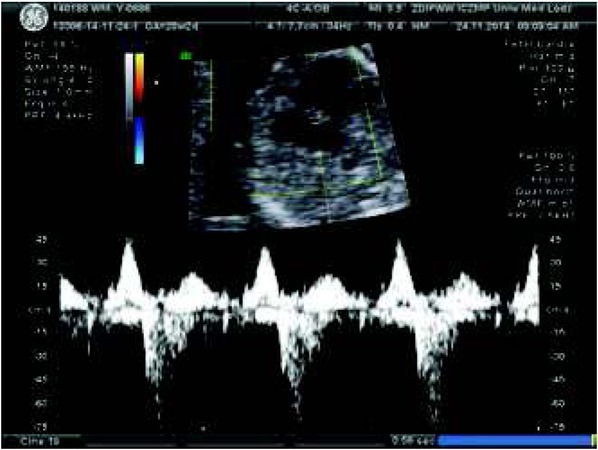
Four chamber view of the fetal heart at 20th week of gestation with fetal tricuspid valve regurgitation – at the time of detection of the congenital heart defect. Ryc. 6. Obraz 4 jam serca płodu w 20 tyg. ciąży z falą niedomykalności zastawki trójdzielnej – wykrycie wady serca.

**Fig. 7:**
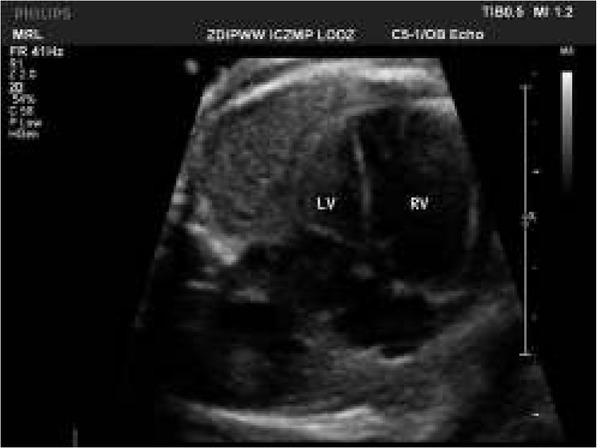
The same patient at 38th weeks of gestation with cardiomegaly, abnormal heart axis, and beginning of fetal congestive heart failure which requires trans-placental therapy for safe pregnancy continuation (fetal echocardiography monitoring and therapy is presented in Table VI). Ryc. 7. Ten sam pacjent w 38 tygodniu ciąży z kardiomegalią, nieprawidłową osią serca, z objawami zaczynającej się niewydolności krążenia wymagającej terapii przezłożyskowej celem bezpiecznego kontynuowania ciąży (opis monitorowania tabela VI).

In the second half of pregnancy, the fetus with a malformation may be monitored using ultrasound during the next few weeks of pregnancy (Figure 6, 7).

Consultations with pregnant women on the mode of delivery and proceedings with the newborn in the middle of gestation is slowly being given up, as in the majority of cases such questions may be answered by the analysis of fetal development only in the second half of pregnancy and more precisely within weeks or even days predating delivery.

Monitoring of a fetus with a cardiac malformation detected optimally in the middle of the pregnancy consists of serial echocardiographic examinations, including several measurable parameters, and of qualitative assessment. The aim of this procedure is to assess the efficiency of fetal circulation, for instance by using the Cardiovascular Profile Score (CVPS) (Table II) [12]. Obtaining 10 points on the CVPS means the absence of any haemodynamic alteration either in the fetus with a normal heart or in the fetus with hypoplastic left heart syndrome. It should be remembered, however, that a fetus who obtained 10 points on the CVPS scale, and in whom disturbances in haemodynamics were observed in the next few weeks, may demonstrate a decrease on the CVPS scale to the level of 6-8 points. In cases with 5 < points on the CVPS, the demise of the fetus or newborn should be expected.

**Table II:** Cardiovascular profile score. Tabela II. Ocena stanu wydolności układu krążenia płodu (CVPS).

Hydrops	None (2 points)	Ascites or Pleural or pericardial effusion *Wysięk w jamie otrzewnowej/Wysięk w jamie opłucnowej/Wysięk w osierdziu* (minus 1)	Skin edema (minus 2) *Obrzęk powłok*
Venous Doppler (Umbilical vein Ductus venosus) *Przepływy żylne: Żyła pępowinowa Przewód żylny*	Normal **(2 points)**	Umbilical vein Normal Ductus venosus reversal flow (**minus 1**)	Pulsation in umb vein (**minus 2**) *Pulsacja w przewodzie żylnym oraz w żyle pępowinowej*
Heart Size assessment *Wielkość serca Ha/Ca*	<0.35 (**2 points** )	0.35-0.5 (**minus 1)**	0.5 <0.2 (**minus 2**)
Cardiac Function *Funkcja serca*	Normal flow TV and MV, biphasic *Przepływy przez z. trójdzielną i mitralna dwufazowe* SF RV/LV >28% **(2 points)**	Holosystolic Tricuspid regurgitation *Holosystoliczna niedomykalność z. trójdzielnej* SF RV/LV <28% (**minus 1)**	TV and MV regurgitation *Holosystoliczna* or monophase *niedomykalność* flow *z. trójdzielnej i z. mitralnej lub monofazowe przepływy* **(minus 2**)
UMB artery flow *Przepływ w t. pępowinowej*	Normal (**2 points**)	No diastolic flow *Brak przepływu w rozkurczu* (**minus 1)**	Reversal flow *Przepływ wsteczny w rozkurczu* (**minus 2**)

In some cases of a threat to the life of the fetus, fetal therapy should be considered – either “through the placenta”, where therapeutics is delivered to the pregnant woman either per os or intravenously, or through the umbilical cord, where therapeutics is delivered by cordocentesis. It is also feasible to deliver therapeutics directly to the fetal buttock. Digoxin is the most frequently used therapeutic drug [13]. Before the decision to deliver a drug to a pregnant woman, her cardiological status should be checked and the rule “*primum non nocere”* is recommended.

In some cases of cardiac malformations, when the haemodynamic state of the fetus is exacerbated and polyhydramnios is observed, several procedures should be considered – i.e. the removal of excess amniotic fluid or the delivery of albumins directly to the fetus, percutaneous balloon valvuloplasty (aortic or pulmonary) or dilatation of foramen ovale. All those procedures are plagued by an increased uterine contractility after the 28th week. Technically, the procedure may be successfully performed but if premature delivery occurs 10-14 days afterwards, it is treated as a complication of intrauterine intervention. Premature delivery in a newborn with a cardiac malformation deprives him or her of the chance to leave the hospital early in up to 60% cases, according to our data from 2017 [14]. The goal is that a pregnancy encumbered with fetal heart malformation should optimally continue to natural delivery at term, with a birth weight >3000 g and a good Apgar score, in a prenatal cardiology center, which collaborates with obstetrical, neonatology, pediatric cardiology and cardiac surgery units.

Whether delivery should be vaginal or by Cesarean section is dependent on the status of the pregnant woman and fetus. Echocardiographic monitoring in the second trimester of pregnancy should be performed between the 20th and the 32nd week every 4^th^ week, then every three weeks (between the 32th and the 35^th^ week), every second week (between 36^th^ and the 38^th^ week) and finally, every week, or even more frequently depending on the cardiac pathology detected. It is most important to observe the status of foramen ovale, the development of pulmonary vessels and flow through the ductus arteriosus.

Independently of echocardiographic monitoring of the fetal heart, the pregnant woman remains under the care of an obstetrician, while a perinatal cardiologist serves as a consultant. For instance, if we deal with fetal cardiac malformation in the form of the common atrio-ventricular canal or double outlet right ventricle, with normal fetal biometry and CVPS 10 and a stable status of the fetus between the 20^th^ and the 36^th^ week of pregnancy, the number of fetal echocardiographic examinations performed in the second half of pregnancy should amount to 2 or 3. The fetus may be born naturally (provided there are no other obstetrical problems, i.e. placenta praevia) and a neonatologist and obstetrician should be present at the delivery.

If the stenotic aortic valve is present in a fetus, first with 20 mmHg gradient (at the 24^th^ week), and 30 mm of Hg gradient (at the 28^th^ week), and 40 mm of Hg gradient (at the 34^th^ week), echocardiographic monitoring close to term is necessary, because with a gradient in the range of 60-70 mm of Hg before delivery, the newborn would require balloon valvuloplasty a few hours after delivery. The earliest procedure at our hospital was performed 2 hours after delivery. Thus, in such cases the time of Cesarean section and availability of a room to perform valvuloplasty should be coordinated in advance, before the time of birth.

Several years ago, we suggested a new classification of fetal cardiac malformations, useful in prenatal cardiology [15, 16]. The old type classification is based on the anatomical details of the fetal heart and still is very important. For years the most frequent type of fetal heart defect in Poland has been Hypoplastic Left Heart Syndrome (Table III). However, what is important in fetal heart defects is not only the anatomy but also the fetus’ haemodynamic status and prognosis for the newborn, just after birth. Therefore, a new classification for the types of fetal heart defects at the end time of gestation is recommended, as well as the composition of the team present during delivery (Table IV). For instance, in case of fetal cardiac malformation in the form of transposition of great arteries with broad foramen ovale and broad patent ductus arteriosus, the newborn may be delivered naturally and he or she would not be in good clinical condition for several hours after delivery. In contrast, a fetus with the same malformation with restricted foramen ovale and premature closure of ductus arteriosus should be treated differently. Such a situation demands a planned Cesarean Section and urgent Rashkind procedure.

**Table III:** The most frequent prenatal congenital heart defects in the National Registry for Fetal Cardiac Problems in the years 2017, 2016 and 2015 (www.orpkp.pl) . Tabela III. Najczęstsze wady serca płodu wg Ogólnopolskiego Rejestru Problemów Kardiologicznych Płodów w latach 2017, 2016 and 2015 (www.orpkp.pl).

2017	
Hypoplastic left heart syndrome	56
Tetralogy of Fallot	46
VSD	46
Complete transposition of great arteries (IVS) – d TGA	43
AVSD: atrial & ventricular septal defect	42
2016	
Hypoplastic left heart syndrome	93
VSD	76
Complete transposition of great arteries (IVS) - d TGA	60
AVSD: atrial & ventricular septal defect	58
Tetralogy of Fallot	57
2015	
Hypoplastic left heart syndrome	76
AVSD: atrial & ventricular septal defect	71
Tetralogy of Fallot	61
Complete transposition of great arteries (IVS) - d TGA	58
VSD	50

**Table IV:** Classification of fetal cardiac defects from prenatal cardiology point of view. Table IV. Podział wad serca z punktu widzenia kardiologii prenatalnej.

	Recommended way of delivery *Zalecany sposób porodu*	Recommended team in delivery room *Optymalny zespół na Sali porodowej*	Recommended neonatal care *Postępowanie z noworodkiem*
CHD severe, not urgent (for cardiac surgery in 1^st^ year of life ex. common atrio-ventricular canal) *Wady serca ciężkie NIEPILNE (do operacji w 1 roku życia, np. wspólny kanał przedsionkowo-komorowy)*	Vaginal delivery *Siłami natury*	Obstetrician, neonatologist, midwife *Położnik, położna, neonatolog*	Neonatal care the same as in a healthy neonate *Takie samo jak w przypadku porodu zdrowego noworodka*
CHD severe for neonatal cardiac surgery (d-TGA) *Wady serca ciężkie PILNE (do operacji w okresie noworodkowym), (np. d-TGA)*	Vaginal delivery *Siłami natury*	Obstetrician, neonatologist, midwife *Położnik, położna, neonatolog*	I.v. line for prostin infusion, neonatal echo and pediatric cardiologist counselling schedule *Założenie drogi dożylnej i wlew z prostinu, wczesne echo i planowa konsultacja kardiologa dziecięcego*
CHD severe and critical (for urgent postnatal balloon valvuloplasty) *Wady serca ciężkie KRYTYCZNE (do pilnej plastyki balonowej po urodzeniu)* e.g.: critical aortic stenosis *np. krytyczna stenoza aortalna)*	Cesarean Section *Cięcie cesarskie*	Obstetrician, neonatologist, midwife Interventional Cardiologist Pediatric *Położnik, położna, neonatolog, kardiolog inwazyjny*	I.v. line for prostin infusion, neonatal echo just after birth and urgent heart catheterisation *Założenie drogi dożylnej u noworodka, wlew z prostinu, echo na Sali porodowej, transport do Sali cewnikowań serca*
CHD: most severe expected fetal or neonatal demise For instance: Severe Ebstein S with lung hypoplasia *Wady serca najcięższe (spodziewany zgon płodu/noworodka) Np. Z. Ebsteina z hipoplazją płuc*	Obstetrical and maternal indications *Wg wskazań położniczych i matczynych*	Obstetrician, neonatologist, midwife *Położnik, położna, neonatolog*	Special delivery room, isolated from other healthy deliveries and comfort care for neonate with special attention for the family *Specjalne pomieszczenie odizolowane od porodów zdrowych noworodków zapewniające komfort i intymność dla rodziców i noworodka*

In Poland, like in other countries, the most frequent cardiac malformation is left heart hypoplasia [11]. Among the ftve most frequent fetal heart malformations in Poland are Fallot syndrome and transposition of the great arteries, while in foreign statistics it is septal defect, including common atrioventricular canal. Those differences reflect both the high level of obstetric screening and basic fetal examination in Poland, which, in turn, reflects the appropriate competences of the physicians awarded the Certificate of the FETAL HEART Examination (the list of such certificates is on www.orpkp.pl).

In the Lodz center, in addition to the screening of the fetal heart, we launched a new classification taking into account prenatal cardiology rules [15], which is similar to the American risk-stratified care of newborns with congenital heart disease determined by fetal echocardiography but was introduced earlier [16]. This classification enables non-specialists (i.e. obstetricians, neonatologists, nurses, midwifes and parents) to prepare for delivery and to follow-up on the newborn in the first days afterwards (Table IV).

In practice, we also encounter situations where cardiac malformation is diagnosed in the third trimester in a fetus developing normally in the first and second trimester. In such cases, estimation of the cardiac structure and haemodynamic status is much more difficult than in cases diagnosed and known since the middle of the pregnancy.

**Table V:** The most frequent types of prenatal cardiac diagnoses. Tabela V. Najczęstsze prenatalne problemy kardiologiczne.

➢ Normal Heart anatomy + Normal Heart Study *Prawidłowa budowa serca płodu + prawidłowe przepływy wewnątrzsercowe*
➢ Normal Heart Anatomy + Functional abnormalities (for instance Tricuspid valve regurgitation) *Prawidłowa budowa serca płodu + nieprawidłowe przepływy wewnątrzsercowe (np. holosystoliczna niedomykalność z. trójdzielnej)*
➢ Normal Heart Anatomy + Myocardial Hypertrophy *Prawidłowa budowa serca + hipertrofia mięśnia serca*
➢ Normal Heart Anatomy + Congestive Heart Failure *Prawidłowa budowa serca + niewydolność krążenia płodu*
➢ Normal Heart Anatomy + Arrhythmia or Fetal Heart Block *Prawidłowa budowa serca + zaburzenie rytmu serca lub przewodnictwa*
➢ Normal Heart Anatomy + Thymus hypoplasia *Prawidłowa budowa serca + hipoplazja grasicy*
➢ Congenital Heart Defect + Normal intracardiac blood flows *Nieprawidłowa budowa serca + prawidłowe przepływ wewnątrzsercowe*
➢ Congenital Heart Defect + Abnormal intracardiac blood flows *Nieprawidłowa budowa serca + nieprawidłowe przepływ wewnątrzsercowe*
➢ Congenital Heart Defect + Extracardiac defect *Nieprawidłowa budowa serca + wada pozasercowa*
➢ Congenital Heart Defect + Abnormal fetal growth (too small for gestational age) *Nieprawidłowa budowa serca + nieprawidłowy wzrost płodu (za mały w stosunku do wieku ciążowego)*
➢ Congenital Heart Defect + Abnormal fetal growth (too big for gestational age) *Nieprawidłowa budowa serca + nieprawidłowy wzrost płodu (za duży w stosunku do wieku ciążowego)*
➢ Congenital Heart Defect + Abnormal fetal karyotype *Nieprawidłowa budowa serca + nieprawidłowy kariotyp płodu*
➢ Congenital Heart Defect + Thymus hypoplasia *Nieprawidłowa budowa serca + hipoplazja grasicy*

This is due to the poor penetration of ultrasounds, which makes it difficult to obtain legible pictures. A prenatal cardiologist must obtain appropriate images, interpret them correctly and obtain a clear acoustic signal from different points of the fetal heart auscultation. The latter is obstructed by the position of the fetus, calcification of the fetal bones, good development of fetal lungs, position of the placenta and the pregnant woman’s weight over 90 kg. In both cases, appropriate prenatal diagnosis, estimation of the fetus’ haemodynamic status, recommendation for delivery and predictions for the newborn are much more difficult and could be less precise.

It could also happen that a newborn is born with cardiac malformation despite several screening ultrasonographic exams, blood tests and biochemical results within normal limits; such a situation is unexpected for both the parents and medical personnel and the diagnosis of cardiac malformation remains in the hands of a neonatologist and paediatric cardiologist. Transportation of a newborn with cardiac malformation not diagnosed prenatally should be regarded as a failure of prenatal medicine. In such cases as common atrio-ventricular canal, delayed diagnosis does not have any consequences for the newborn, apart from stress for the mother and father, but in cases of critical stenosis of the aorta, delivery not taking place in a neonatological-cardiological unit, transportation of the newborn and delayed valvuloplasty may mean a cardiosurgical procedure. It may also result in neurological complications, long lasting rehabilitation, and finally, the newborn’s demise.

In the prenatal period, cardiological problems are not merely malformations, or cardiac fetal insufficiency. Fetal arrhythmias may also be present: (extrasystole, tachycardia or atrio-ventricular blocks). These are problems typical for multiple fetal pregnancies, i.e. the twin-twin transfusion syndrome, conjoined twins and haemodynamic complications of twin demise in multiple pregnancies [17, 18].

The cardiological status is crucial in cases of extracardiac malformation, like duodenal atresia, arthrogryposis or renal malformations. Prognosis in cases of extracardiac malformations often depends on the cardiac status.

Obstetricians and perinatologists should be aware of possible fetal cardiac problems, particularly in cases of abnormal CTC and atypical images of flows by Doppler in peripheral fetal vessels. Better interpretation and prevention of premature deliveries brings better results; in contrast relying on false results from less precise examinations makes prognosis worse. Investment in prenatal diagnosis and prenatal therapy also has a financial dimension because better results may be obtained in a shorter time of hospital stay. As we demonstrated for a newborn with critical aortic stenosis diagnosed prenatally, normal delivery at the 39th week of gestation, and balloon valvuloplasty performed on the first day of life, the newborn was hospitalized at the intensive care unit for 2 days and altogether for 18-20 days in the hospital. In contrast, a newborn with the same type of cardiac malformation but without prenatal diagnosis and premature delivery and unsuccessful balloon valvuloplasty at 8 days, had a cardiosurgical procedure, was hospitalized at the intensive care unit and discharged after 3 months of hospitalization [19].

**Table VI:** An example of fetal echocardiography monitoring in a fetus with a severe planned cardiac defect (Absent Pulmonary Valve) and introduction of fetal – transplacental treatment at the time of deterioration to prepare the fetus for delivery in a tertiary center and give him a chance to survive. Vaginal delivery > 37^th^ week of gestation, birth weight 2900g, Apgar 9. For 5 days the newborn was in a good clinical condition and required intubation on the 6^th^ day after birth. She had total cardiac surgery correction on the 25^th^ day of postnatal life – team of Prof. Jacek Moll in Łódź - and was discharged home on the 43^rd^ day of postnatal life. Tabela VI. Przykład monitorowania parametrów echokardiograficznych u płodu z ciężką planową wadą serca, (braku zastawki t. płucnej) i wprowadzenia terapii płodu w okresie zagrożenia, celem przygotowania płodu do porodu w ośrodku referencyjnym i stworzenia warunków do przeżycia dla noworodka. Poród siłami natury >37 tyg. ciąży, masa ciała 2900 g, Apgar 9, 5 dni noworodek wydolny oddechowo, intubacja w 6 dobie, korekcja całkowita w 25 dobie – zespół prof. Jacka Molla, wypis do domu w 43 dobie.

Nr of fetal echo exams	No. 1	No. 2	No. 3	No. 4	No. 5	
WKs of gest	18.6/18	21.5/21.5	27.3/27.3	34.3/34.4	37.3/37.3	
Ha/Ca	0.3	0.3	0.45	0.45	0.46	
AP	17	20	30	40	40mm	
AFI	13	10	10	12	9	
Pulmonary trunk (mm)	5	7	8	9.9	10	
Pulmonary branches (mm)	6	7.5	10	13 and 13.9	16	
CVPS	10	10		8	10	10
Treatment				Digoxin i.v. Steroids i.m	Digoxin p.o. Steroids i.m. Oxygen 4 x a day	Digoxin Oxygen

Taking into account all the above-mentioned problems, state consultants and teams writing board specialization tests should be asked whether there is enough teaching of prenatal cardiology and whether it should perhaps be more broadly covered in the undergraduate teaching of medical students [20].

Institutional bases of prenatal cardiology were created some 15 years ago in Poland within the framework of the Ministry of Health “Cardio-Prenatal” program headed by state consultant Prof. Wanda Kawalec. The Polish Registry of Fetal Cardiological Problems was created (www. Orpkp.pl [21]). Within this Registry, some 7000 records were stored up to 2017 and a group of 70 physicians were awarded certificates as FETAL HEART Examination specialists. It was also stressed that prenatal cardiology is becoming an independent branch of medicine including elements of obstetrics, neonatology, pediatric cardiology, radiology and genetics.
